# Refining the genetic structure and relationships of European cattle breeds through meta-analysis of worldwide genomic SNP data, focusing on Italian cattle

**DOI:** 10.1038/s41598-020-71375-2

**Published:** 2020-09-03

**Authors:** Salvatore Mastrangelo, Marco Tolone, Slim Ben Jemaa, Gianluca Sottile, Rosalia Di Gerlando, Oscar Cortés, Gabriele Senczuk, Baldassare Portolano, Fabio Pilla, Elena Ciani

**Affiliations:** 1 Dipartimento Scienze Agrarie, Alimentari e Forestali, University of Palermo, 90128 Palermo, Italy; 2 Laboratoire des Productions Animales et Fourragères, Institut National de La Recherche Agronomique de Tunisie, Université de Carthage, 2049 Ariana, Tunisia; 3 Dipartimento Scienze Economiche, Aziendali e Statistiche, University of Palermo, 90128 Palermo, Italy; 4 Departamento de Produccion Animal, Universidad Complutense de Madrid, 28040 Madrid, Spain; 5 Dipartimento di Agricoltura, Ambiente e Alimenti, University of Molise, 86100 Campobasso, Italy; 6 Dipartimento di Bioscienze Biotecnologie e Biofarmaceutica, University of Bari, 70124 Bari, Italy

**Keywords:** Inbreeding, Population genetics, Animal breeding

## Abstract

The availability of genotyping assays has allowed the detailed evaluation of cattle genetic diversity worldwide. However, these comprehensive studies did not include some local European populations, including autochthonous Italian cattle. In this study, we assembled a large-scale, genome-wide dataset of single nucleotide polymorphisms scored in 3,283 individuals from 205 cattle populations worldwide to assess genome-wide autozygosity and understand better the genetic relationships among these populations. We prioritized European cattle, with a special focus on Italian breeds. Moderate differences in estimates of molecular inbreeding calculated from runs of homozygosity (*F*_ROH_) were observed among domesticated bovid populations from different geographic areas, except for Bali cattle. Our findings indicated that some Italian breeds show the highest estimates of levels of molecular inbreeding among the cattle populations assessed in this study. Patterns of genetic differentiation, shared ancestry, and phylogenetic analysis all suggested the occurrence of gene flow, particularly among populations originating from the same geographical area. For European cattle, we observed a distribution along three main directions, reflecting the known history and formation of the analyzed breeds. The Italian breeds are split into two main groups, based on their historical origin and degree of conservation of ancestral genomic components. The results pinpointed that also Sicilian breeds, much alike Podolian derived-breeds, in the past experienced a similar non-European influence, with African and indicine introgression.

## Introduction

The domestication of cattle approximately 10,000 years ago changed the social and economic life of most human populations^1,2^. Genetic drift, together with natural and artificial selection, led to the development of divergent breeds^3^ that differ in several phenotypic traits, including coat color, body size, behavior, and production traits^2^. Until the first industrial revolution in the late eighteenth century, cattle genetic diversity, especially in Europe, was mostly shaped by selection and adaptation to the natural environment of local breeds^4^. However, during the last two centuries, locally well-adapted but, less successful populations were progressively replaced by commercial, highly productive breeds with the aim of increasing profitability for farmers^5^. Cattle are the species with the highest number of breeds at risk of extinction, indicating that cattle genetic diversity is gradually being depleted^6,7^.

The availability of genotyping assays has made it possible to conduct a detailed evaluation of cattle genetic diversity globally^2,8–13^. According to these studies, the European cattle gene pool is mainly of *Bos taurus taurus* origin. There are a few exceptions to this rule, as in the case of Turkish and some Italian beef breeds (Chianina, Romagnola, and Marchigiana) which show evidence of mixed *B. t. taurus* and *B. t. indicus* ancestries. Similarly, Iberian cattle were shown to have African taurine introgression^10,14^, while the Italian Podolica breed is an ancient cross-breed in which indicine introgression has occurred^14^. However, these comprehensive studies did not include several local European cattle characterized in recent investigations^7,15,16^, including many Italian cattle breeds^17^. As a consequence of this gap, the relationships between these cattle and other breeds existing worldwide have not been clarified and addressed, and a comprehensive description of the distribution of the diversity of present-day European cattle is still lacking.

In the present study, we collected a large genomic dataset containing newly generated genotyping data from several European cattle, especially Italian breeds. We aimed to contextualize the genetic variation of the newly added breeds with respect to domesticated bovid populations worldwide. Specifically, we determined the population structure and genetic relationship of the new breeds with other populations from around the world.

## Results and discussion

**Figure 1:**
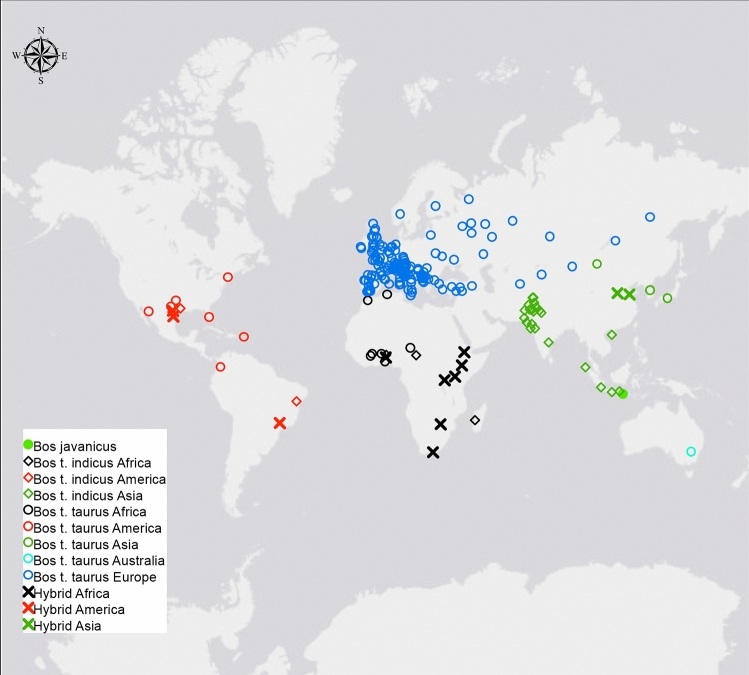
Geographic distribution of the 205 different domesticated bovid breeds on the world map.

Numerous studies have been undertaken to describe the phylogeny of cattle populations on a global scale using high-throughput genotyping assays^2,8,10,11,13,15^. However, the genetic relationships with some local breeds that were not previously included in these global studies remain ambiguous. In this study, we assembled a large cattle dataset to provide a closer examination of inbreeding, admixture patterns, and genetic relationships among cattle worldwide, with a special focus on Italian breeds. After quality control, the merged dataset used in the analyses consisted of 3,283 individuals from 205 domesticated bovid populations (*n* = 1 *B. javanicus*, *n* = 27 *B. t. indicus*, *n* = 165 *B. t. taurus*, and *n* = 12 hybrids) (Table S1, Table 1, and Fig. 1) genotyped on 23,313 SNPs.

**Table 1:** Number of individuals (N ind) and populations (N pop) by sub-species and geographic origin.

	Asia		Americas		Africa		Australia		Europe	
	N ind	N pop	N ind	N pop	N ind	N pop	N ind	N pop	N ind	N pop
Hybrids	9	2	60	3	90	7	–	–	–	–
*B. t. indicus*	235	22	40	2	60	3	–	–	–	–
*B. t. taurus*	25	3	99	7	143	7	4	1	2,498	147
*B. javanicus*	20	1	–	–	–	–	–	–	–	–

### Runs of homozygosity

**Figure 2:**
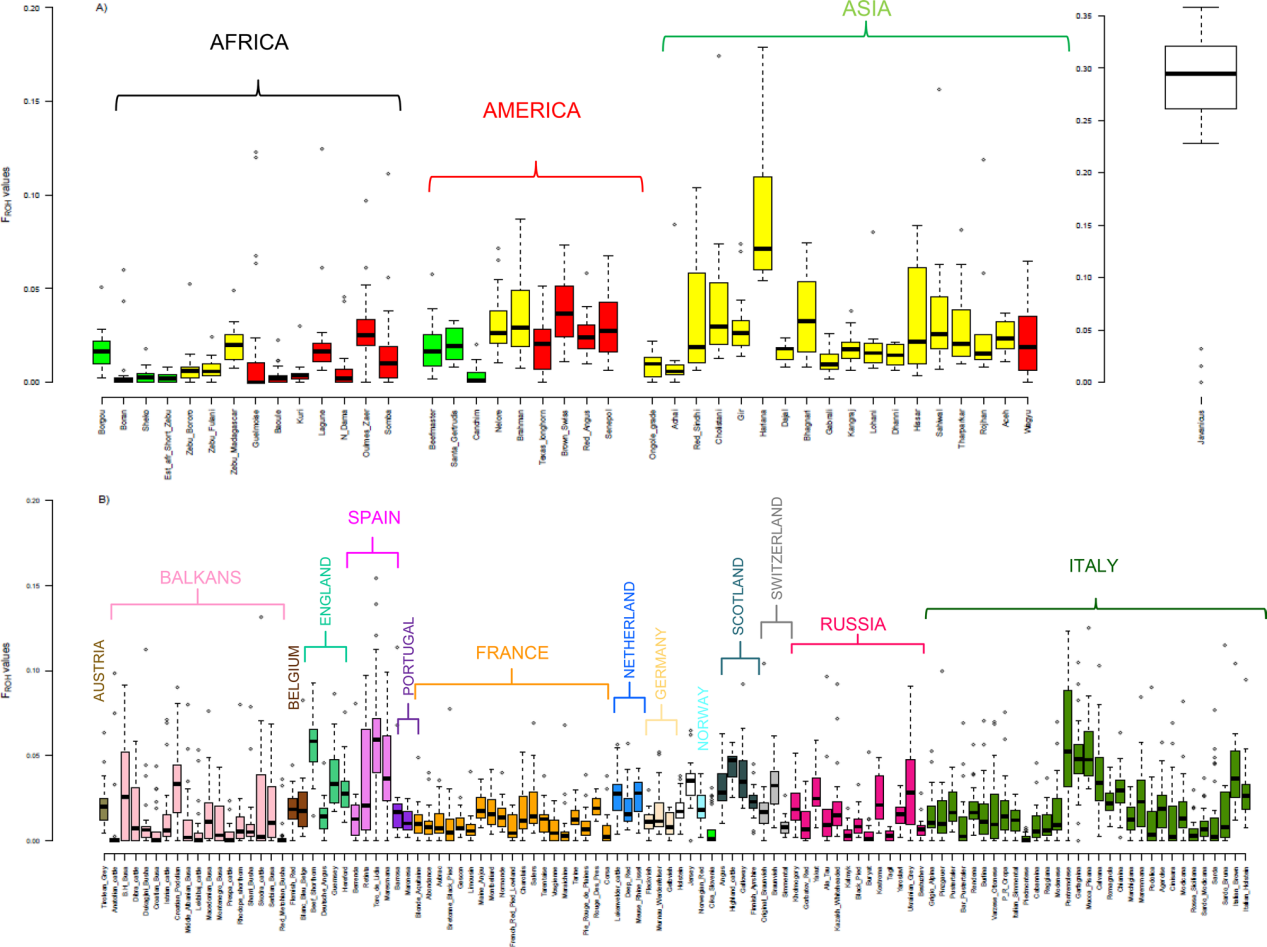
Box plot of the inbreeding coefficients inferred from runs of homozygosity (*F*_ROH_) for all breeds with equal or more than 10 individuals per breed. (**A**) *B. t. taurus* (red), *B. t. indicus* (yellow) and hybrids (green) from Africa, America and Asia, respectively. (**B**) *B. t. taurus* from Europe depicted with different colors based on the geographic origin of the breeds (Austria, Balkans, Belgium, England, Spain, Portugal, France, Netherland, Germany, Holstein and Jersey, Slovenia, Scotland, Switzerland, Russia and Italy).

Currently, ROH-based inbreeding estimates (*F*_ROH_) are one of the most powerful approaches to detect inbreeding effects and conceivably recent population bottlenecks^17^. We estimated *F*_ROH_ to identify genome-wide autozygosity for all populations with a sample size ≥ 10, which led to the retention of 151 populations (2,992 individuals) (Fig. 2), i.e., this was one of the largest and most complete datasets used to date for this type of analysis in cattle. Long ROHs (~ 10 Mb) reflect recent inbreeding (up to five generations ago), while short ROHs (~ 1 Mb) can indicate more distant ancestral effects (up to 50 generations ago) and past selection events^17^. We defined ROHs as tracts of homozygous genotypes > 4 Mb in length, which were related to more ancient inbreeding, occurring from 12.5 generations ago (approximately 75 years ago) to the present day. Except for Bali cattle (*B. javanicus*) (*F*_ROH_ = 0.26) and the Hariana zebuine breed (*F*_ROH_ = 0.10) (Fig. 2A), all populations showed moderate (> 0.05) to low (< 0.05) *F*_ROH_ values (ranging from 0.01 to 0.07). Similarly, a recent SNP-based analysis performed on whole-genome sequencing data from European and African taurines, in addition to four indicine populations, reported that genome ROHs did not vary greatly among the individual cattle studied^14^. The same study reported that several southern European cattle displayed numbers and cumulative lengths of ROHs comparable to that of N'Dama cattle from Africa. Our results confirmed these findings, as we found that African populations (both taurine and zebuine cattle) showed mean *F*_ROH_ values comparable with those of most European breeds.

Notably, we found marked differences in *F*_ROH_ estimates between different European breeds. For instance, Vosgienne, Limousin (France); Tagil, Kalmyk (Russia); Red Metohian Busha, Anatolian cattle (Balkans); and Piedmontese, Reggiana, and Rossa Siciliana (Italy) showed the lowest *F*_ROH_ levels (< 0.02) (Fig. 2B). Breeding strategies based on the minimization of the mean kinship coefficient between animals likely underpin the low *F*_ROH_ values in some Italian (e.g., Piedmontese) and French (e.g., Limousin) breeds. Similarly, high levels of admixture may explain the low *F*_ROH_ values observed within some European (e.g., Balkan cattle) and admixed African populations (e.g., Boran, Sheko, and Guelmoise) that have not undergone breeding programs. Conversely, some European breeds such as Toro de Lidia (Spain), Pontremolese, Garfagnina, and Mucca Pisana (Tuscan region of Italy) showed the highest estimates of molecular inbreeding, with mean *F*_ROH_ values > 0.05. This was most likely due to a reduction in their population sizes and uncontrolled mating of related individuals. Indeed, Pontremolese, Garfagnina, and Mucca Pisana have experienced a population decline during the second half of the twentieth century, resulting in these breeds being classified as endangered^17^. Toro de Lidia is a genetically isolated breed due to the peculiarity of the breeding objectives of this breed (aggressiveness). Moreover, the breed comprises several lineages as a result of different behavioral selection objectives. This resulted in a considerable increase in the average relatedness value within lineages (0.144), a very high score when compared with other cattle breeds^18^. Our observation of high *F*_ROH_ value for Bali cattle suggests that the greater genetic distances from the SNP discovery panel breeds introduce a significant ascertainment bias into autozygosity estimates. In this study, owing to the minimum 4 Mb size imposed on ROH segments, we tried to avoid small autozygous segments so as to reduce ascertainment bias that would lead to overestimation of molecular inbreeding^13^. The data suggest that any potential bias did not appear to affect markedly our results. For its part, the high *F*_ROH_ value (0.10) observed for Hariana cattle (a prominent dual-purpose breed from North India primarily reared for bullock production) can be attributed to a significant deficit in heterozygosity, as previously reported for this breed based on microsatellite data^19^.

Our findings indicate the need for the continued monitoring of inbreeding rates and implementation of breeding strategies that minimize inbreeding, particularly for breeds with limited geographical distribution and small population size.

### Genetic relationship and admixture

To identify the genetic relationships among the 205 cattle breeds belonging to three domesticated subspecies (*B. javanicus*, *B. t. indicus*, and *B. t. taurus*), to refine and extend previous studies, and to provide a new global picture of cattle genetic diversity, we carried out a multidimensional scaling (MDS) analysis, graphically depicted the estimated Reynold’s genetic distances by Neighbor-Net analysis, and inferred ADMIXTURE events. In addition, a subset of data containing only European and Eurasian populations (147 breeds) was analyzed separately to obtain a fine resolution at the local level, with a special focus on Italian cattle breeds.

#### Worldwide level

**Figure 3:**
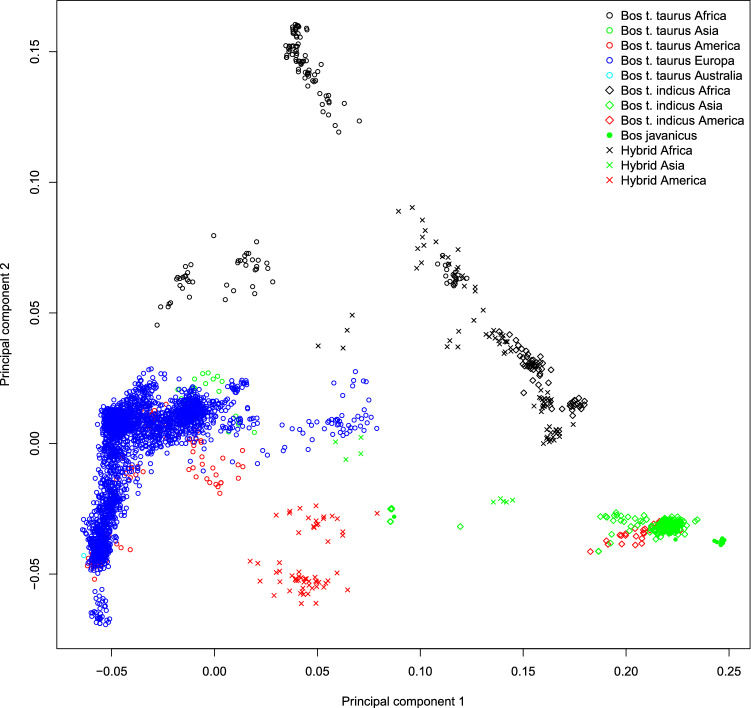
Genetic relationship among the worldwide cattle breeds in this study as inferred by MDS analysis. Points were colored according to the geographic origin of breeds; black (Africa), green (Asia), red (America), blu (Europe) and light blue (Australia). Different symbols are used for the four domesticated bovid (sub)species. The first two components, C1 and C2, accounted for 15.34 and 2.87%, respectively of the total variation.

**Figure 4:**
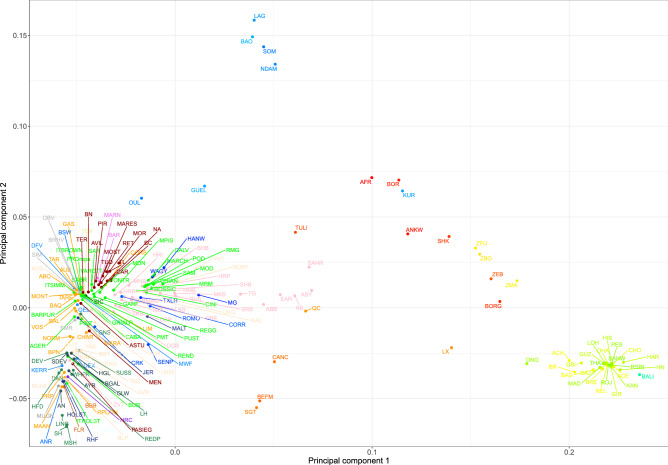
Genetic relationship among the worldwide cattle breeds in this study as inferred by MDS analysis. Each point (per a total of 205) represents breed-average coordinates of eigenvalues of C1 and C2. For full definition of breeds see Table S1.

According to the MDS analysis (Fig. 3), *B. t. indicus* and *B. t. taurus* were separated by the first axis (PC1) with the hybrid breeds spread between the two. These observations are consistent with documented knowledge of cattle history^10,20,21^. The second axis splits African taurine cattle from European, American, Eurasian taurine, indicine, and hybrid populations, suggesting a strong effect of genetic drift in African taurine when compared with Eurasian taurine cattle^13^. Mediterranean breeds clustered together and were closer to the African taurine group than to North European breeds (Fig. 4 and Supplementary Fig. 1). All the American taurine breeds were placed in the European taurine group, while the American indicine breeds (Nelore and Brahman) clustered with Asian indicine populations (Fig. 4 and Supplementary Fig. 1). In this study, as well as also previously reported^2,8,10,11,13,22,23^, cattle genetic diversity can be described as a triangle with vertices represented by (i) West African taurine (Baoulè, Somba, Lagune, and N’Dama), (ii) European, and (iii) indicine populations. Guelmoise (Algeria) and Oulmes-Zaer (Morocco) breeds fell at an intermediate position between South European and West African taurine populations (Fig. 4), thereby confirming the previously reported admixed origin of North African cattle^2,24^. The Anatolian breeds (Anatolian Black, Anatolian cattle, Anatolian Southern Yellow, East Anatolian Red, and South Anatolian Red), together with Turkish Grey cattle, were positioned further from the European breeds and were slightly tilted toward zebu populations, suggesting an indicine influence. The Chinese Qinchuan cattle was placed close to Anatolian breeds (Fig. 4); this observation supports previous results^25^ that inferred a similar admixture pattern for Qinchuan and Turkish cattle breeds, reflecting their similar degrees of zebu introgression.

**Figure 5:**
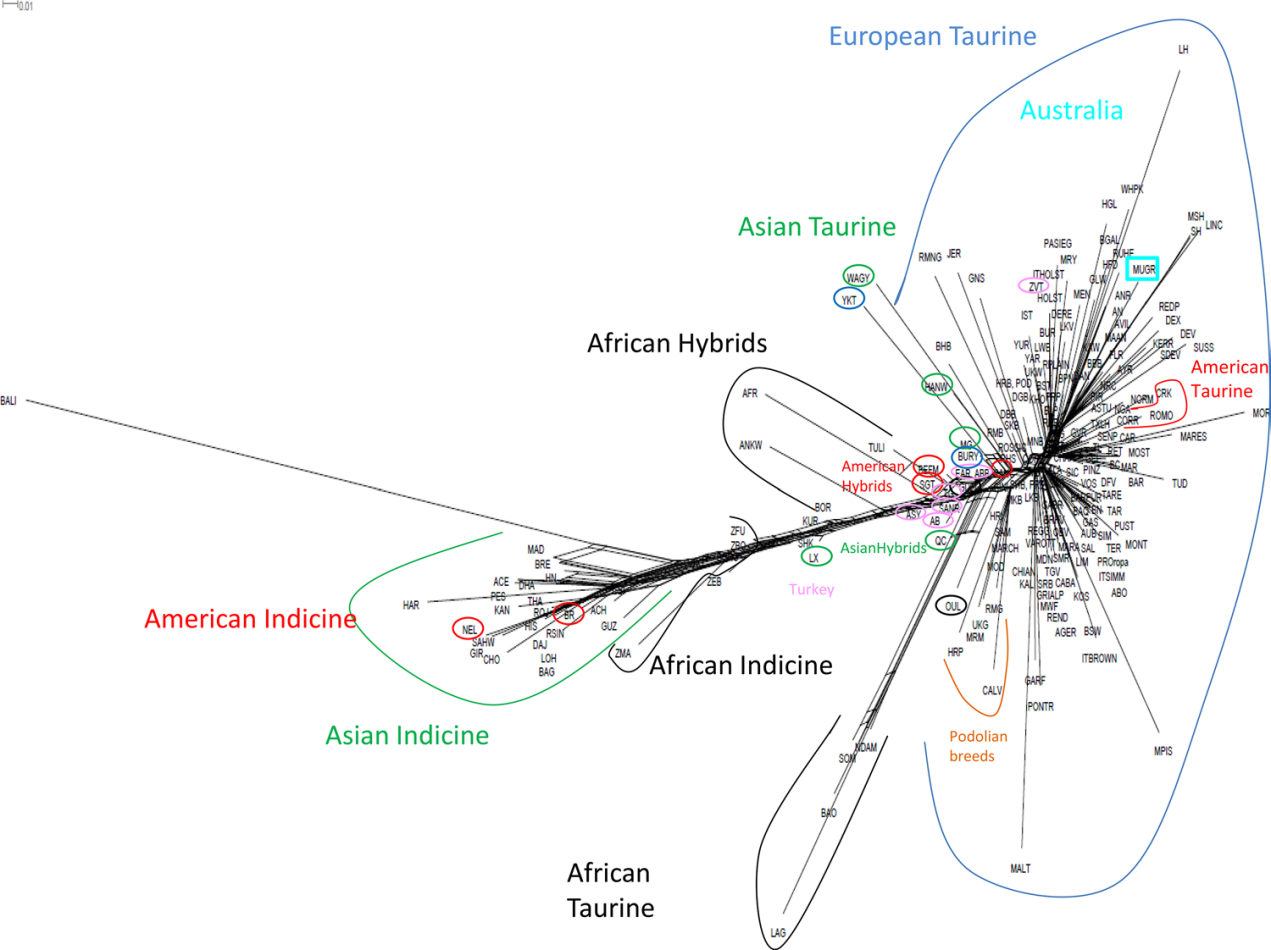
NeighborNet graph based on Reynolds genetic distances for the 205 different domesticated bovid breeds: black (Africa), green (Asia), red (America), blu (Europe) and light blue (Australia). For full definition of breeds, see Table S1.

To provide additional insight into cattle phylogeny, relationships, and patterns of divergence, we constructed a Neighbor-Net network based on Reynolds genetic distances among the 205 cattle populations (Fig. 5). The results were in agreement with those of the MDS plot described above. The graph indicated the separation among Eurasian (including American taurines, to the right of the figure), African taurines, and indicine breeds, with hybrid populations (African, Asian, and American) being found between indicine and Eurasian taurine cattle. Recently, using a different dataset, Pitt et al.^13^ identified two main clusters of hybrids, namely, African hybrids that were closer to African indicine breeds, and American hybrids that were closer to European taurine cattle, with an intermediate position for Asian hybrids. In our study, all the hybrid populations were positioned together in the same area of the network, adjacent to the Turkish breeds. This network also separated the European breeds into four main clusters based on geographic origin: Northern Europe, America, Central-Southern Europe, and the Podolian group. Several breeds that are known to have a relatively small population size (e.g., Maltese) and/or higher degree of inbreeding (e.g., Bali and Mucca Pisana) showed characteristic longer branches, indicative of strong genetic drift or selection pressure. The longest branches reflect the combined effects of ascertainment bias, reproductive isolation, bottleneck, reduced population size, and distinctiveness.

**Figure 6:**
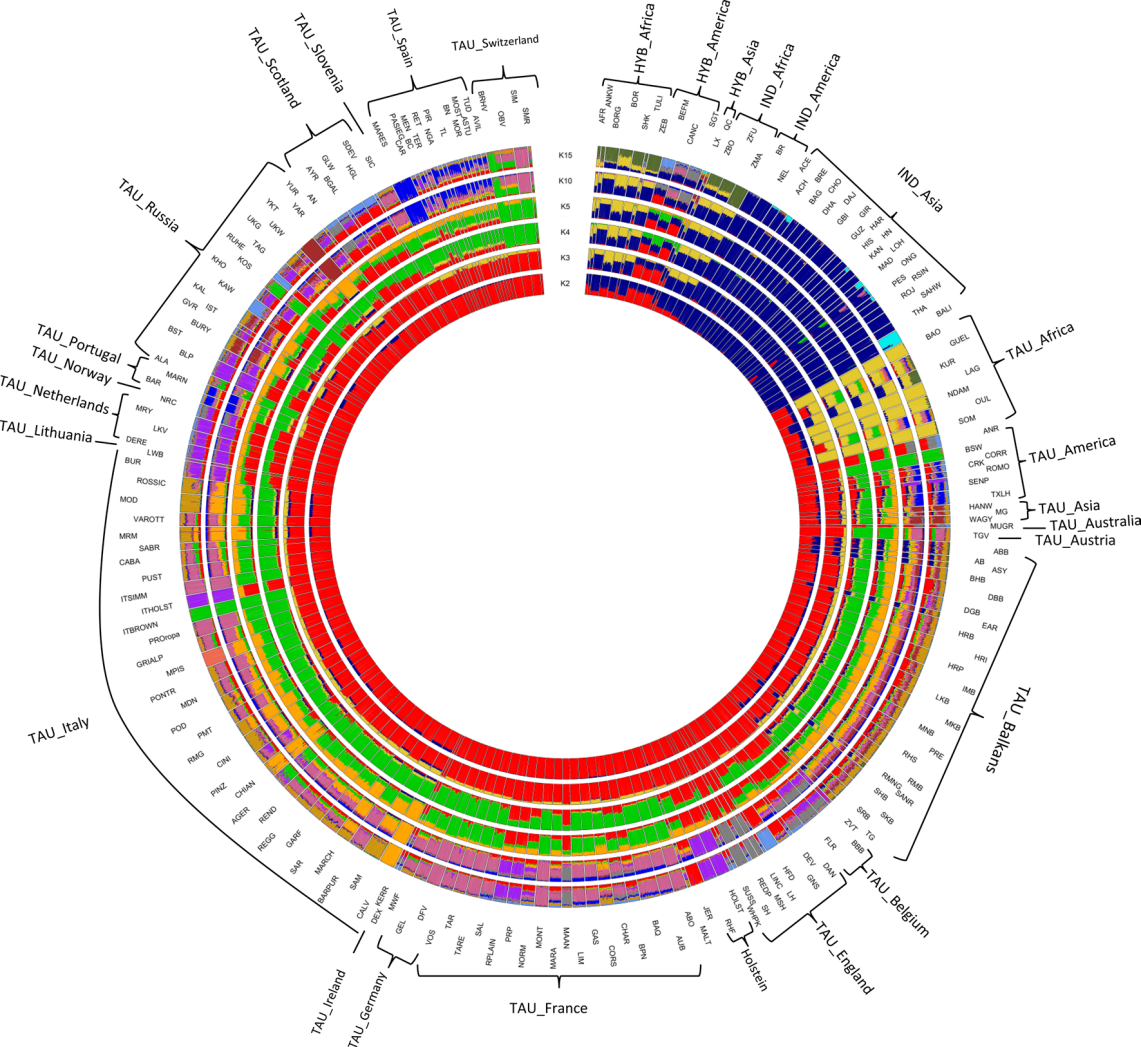
Circular representation of the worldwide population structure for the 205 different domesticated bovid breeds inferred from the ADMIXTURE analysis. The tested numbers of clusters (K) assumed in the total sample were K = 2, 3, 4, 5, 10 and 15.

The ADMIXTURE analysis (Fig. 6) revealed the subdivisions among the domesticated populations, and reproduced the results of the MDS and Neighbor-Net analyses. The population subdivision at K = 2 reproduced the first PCA coordinate by separating *B. t. taurus* and *B. t. indicus* cattle (red and dark blue, respectively). At K = 3, African *B. t. taurus* populations formed a separate cluster (gold). African indicine and African hybrids shared a roughly similar proportion of African taurine and Asian zebuine ancestry, suggesting that elements of taurine descent (gold) may still be present in the genome of African zebuine cattle. The results of the *f3* test highlighted clear signs of African taurine (Lagune, Kuri, and Guelmoise breeds) and African zebuine (ZFU) admixture (Table S2). For values of K from 2 to 5, the indicine and African taurine genomic components were evident in the Balkan and in the American taurine breeds (Fig. 6), consistent with the results of previous studies^10^. Similarly, our results confirmed those of previous studies^15,26^ showing that Russian breeds (Buryat, Kalmyk, Yakut, and Ukrainian Grey) shared ancestry with cattle from Asia. Interestingly, we also detected an African component in Russian cattle that has not been previously described. This result was corroborated by *f3* statistics, which showed evidence for African taurine (Guelmoise and Kuri) and African indicine (ZBO and ZFU) ancestry in several Russian cattle breeds (Table S2).

Within-continent and national substructures were identified when the number of ancestral populations was increased (higher K values). Breeds were progressively assigned to separate clusters. For example, the Holstein group, Marismeña, and Ukrainian Whiteheaded formed distinct clusters at K = 10, while the Mucca Pisana separated from the other breeds at K = 15 (Fig. 6). The results showed that these groups/breeds have a clearly defined genetic identity. Moreover, at K = 15, the split between Asian (dark blue) and African (dark olive green) *B. t. indicus* became clearer. Increasing the number of inferred clusters (K values from 20 to 50) (Supplementary Fig. 2) allowed us to identify highly admixed groups, such as the Balkan group. This result was consistent with their heterogeneous phenotypic characteristics and the lack of controlled mating and phenotype recording^6^. Moreover, it was possible to observe ancestral components that appear to be shared among different groups, such as between the Bali, Balkans, and Russian groups (cyan).

#### A focus on the Italian breeds in a global context

Italian cattle clustered with the other European, Russian, American (Brown Swiss, Angus, Romosinuano, Florida Cracker, Corriente, and Texas Longhorn), and Asian (Wagyu, Hanwoo, and Mongolian breeds) taurine populations (Fig. 4 and Supplementary Fig. 1). European cattle were exported into Asia and evidence of European introgression into these Asian breeds has been documented^9^. Italian breeds (in green) were split into two main groups when plotting the breed-average coordinates of eigenvalues of C1 and C2 (Fig. 4). The first group included the Northern-Central breeds, while the second grouped the Podolian and the Sicilian breeds closest to the zero value of the first principal component.

In the ADMIXTURE plot, at values of K ranging from 2 to 5, the six Podolian (Chianina, Calvana, Marchigiana, Maremmana, Podolica, and Romagnola), Sicilian (Cinisara, Modicana, and Rossa Siciliana), and Sardo-Modicana breeds presented minor contributions from indicine (dark blue) and African taurine (gold) cattle (Fig. 6). African and indicine introgression was previously reported for Maremmana, Chianina, Marchigiana, Podolica^14^, and Romagnola^10^. Our analyses extend these findings to the Sicilian breeds. This result suggests that Cinisara, Modicana, Rossa Siciliana, and Sardo-Modicana, as well as Chianina, Calvana, Marchigiana, Maremmana, Podolica, and Romagnola, have experienced a similar non-European influence. This is evident from the graph in Fig. 5, which shows that the branches comprising the Podolian (Italian and European) and the Sicilian breeds occupy an intermediate position. The relatively short distances between this group and the indicine/taurine hybrids in the Neighbor-Net analysis may indicate the occurrence of past admixture events. Moreover, the significant negative *f*_3_ statistics obtained when Sicilian breeds (Cinisara and Rossa Siciliana) were used as reference populations were indicative of a zebu ancestry (Table S2). A recent study suggested that the influx of *B. t. indicus* ancestry into *B. t. taurus* populations was likely to have been human-mediated and further driven by an abrupt climate change event that occurred approximately 4,200 years ago^27^.

Our results showed that several Italian cattle breeds (Podolian and Sicilian breeds) share indicine ancestry with Balkan cattle (Fig. 6, from K = 10 to K = 15, dark orange-red), suggestive of a common origin of the indicine ancestry in these populations^14^. Additionally, Northern-Italian breeds share ancestry with French and Swiss cattle, reflecting a historical gene flow among these populations. These findings suggested that continental European breeds contributed to this Northern-Italian cattle gene pool. With increasing K clusters, several European breeds (including the Italian ones) are increasingly distinguished. For instance, at K = 50, Calvana, Garfagnina, Romagnola, Pontremolese, and Mucca Pisana breeds separated as distinct breeds (Supplementary Fig. 2). The Piedmontese is an important Italian beef cattle breed for which the inclusion within the Podolian group is still debated. The Piedmontese breed belongs to the cattle breeds of the Northern Italy group, and its ancestral origin includes *B. brachyceros* and a mixture of *B. brachyceros* and *B. primigenius*^28^. Phenotypically, this breed and the Podolian group both have grey coats. However, we did not identify either an indicine or an African taurine component in the genome of this breed via the admixture analysis. Moreover, the Piedmontese was not positioned in the same Neighbor-Net branching containing the Italian and European Podolian breeds (Fig. 5). In a recent study on mitochondrial DNA variants of Podolian breeds, Di Lorenzo et al.^29^ reported that this breed and the other six white Podolian-derived beef cattle breeds had a different ancestral origin, which may explain the results reported in this study. The other Italian breeds showed extensive sharing of genomic components with the European taurine cattle breeds. This analysis indicated that the genetic background of the Italian breeds is remarkably complex, and that the breeds belonging to the Podolian group show a marked genetic distinctiveness when compared with Northern-Central Italian breeds.

#### European level

**Figure 7:**
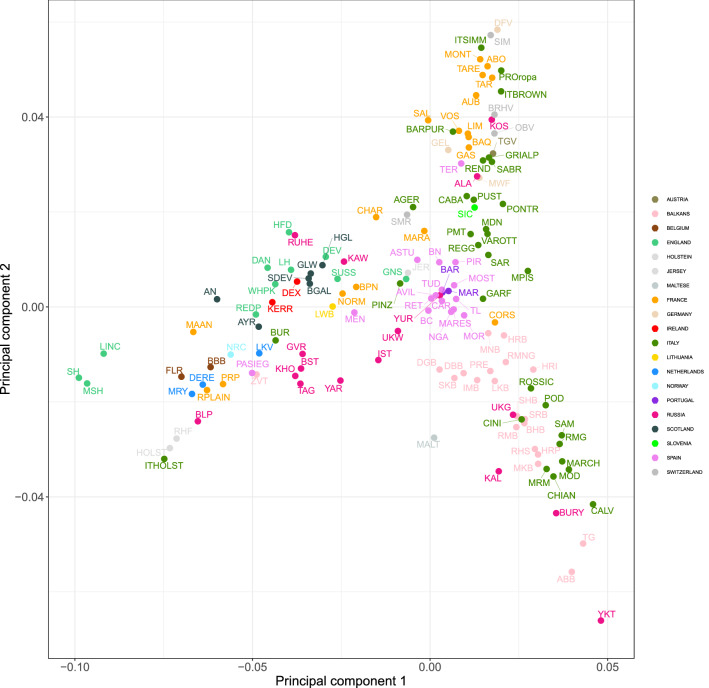
Genetic relationships among the European cattle breeds in this study as inferred by MDS analysis. Each point (N = 147) represents breed-average coordinates of eigenvalues of C1 and C2. For full definition of breeds, see Table S1.

To gain a better insight into the genetic relationships among European breeds, we performed the previously described analyses on the 147 European taurine breeds of our dataset. The MDS analysis indicated that the genetic diversity of European cattle was represented by a gradient of distribution along three main directions, with vertices represented by (i) Anatolian breeds and the Yakut; (ii) Red Pied cattle breeds (Fleckvieh, Simmental, Montbeliard, Abondance, and the local Italian breed Pezzata Rossa d’Oropa); and (iii) Lincoln Red, Shorthorn, and Holstein (Fig. 7). This result reflected the known history and formation of the analyzed breeds. The Anatolian breeds are admixed with African taurine introgression, whereas the Shorthorn populations are the most distinct group of European cattle^10^. Yakut, belonging to the Russian cattle breeds that share ancestry with Asian taurines, has been reported to be a divergent breed^15,26^. We further confirmed this in our study, where Yakut was placed near the Asian taurine in the worldwide Neighbor-Net graph (Fig. 5). The third vertex shows a cluster of cattle (Red Pied or Simmental breeds) with the same aptitude (dual purpose), a common genetic origin, and geographical proximity. Simmental is an old cattle breed widely distributed globally, and is believed to be the result of a cross between German cattle and a small Swiss indigenous breed^30^. Previous studies have shown that there is a weak genetic relationship between European and Simmental breeds^2,31,32^.

**Figure 8:**
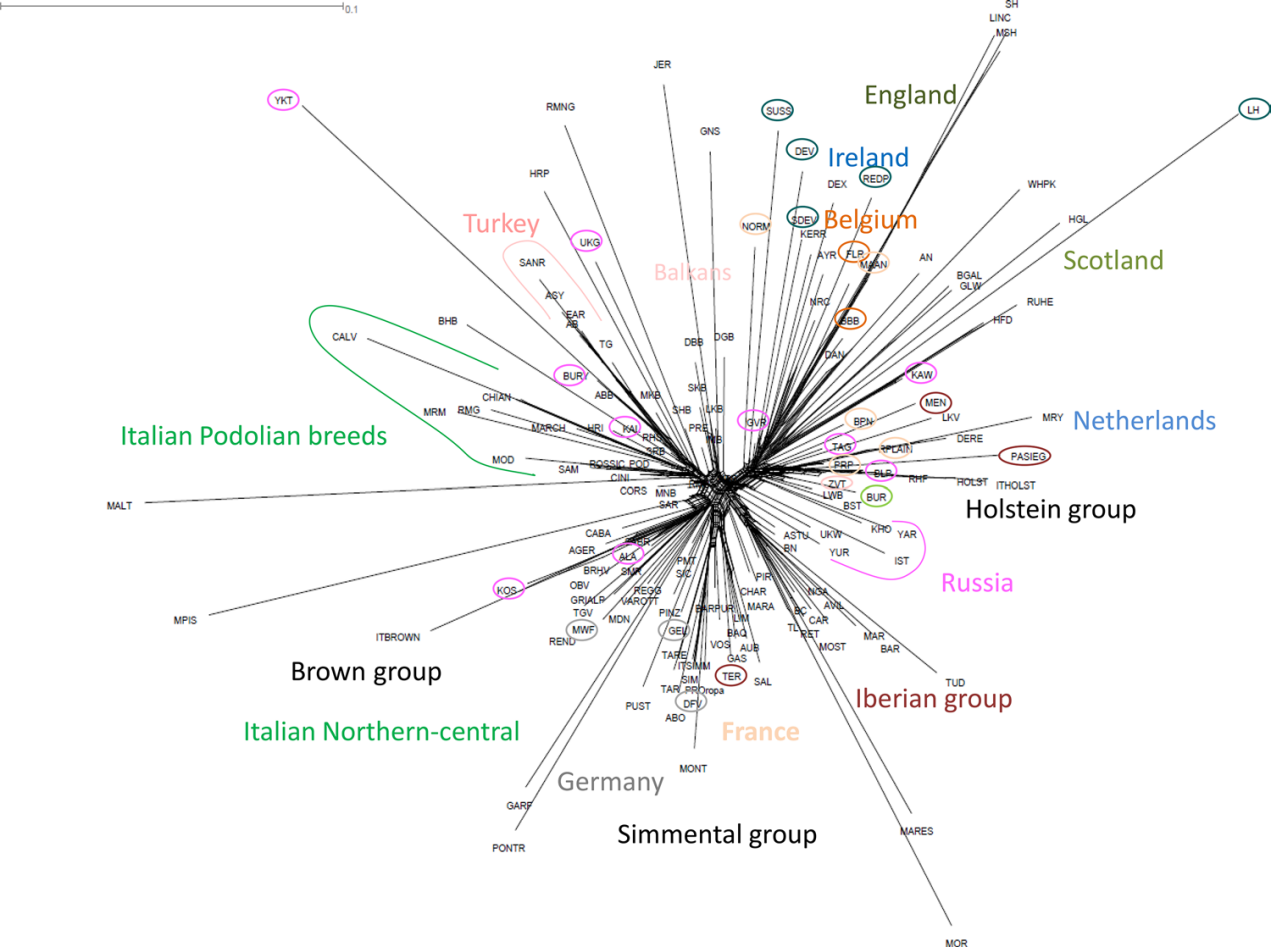
NeighborNet graph based on Reynolds genetic distances between 147 European cattle breeds. For full definition of breeds, see Table S1.

Similarly, the Neighbor-Net phylogenetic network (Fig. 8) showed that European populations clustered according to their genetic origin and/or geographical proximity to clades broadly corresponding to (i) North European breeds (Belgium, Ireland, England, Scotland, and the Netherlands) and the Holstein group; (ii) Iberian group; (iii) French, German, and Simmental group; (iv) Northern-Central Italian and Brown group; (v) Italian Podolian and Turkish breeds; and (vi) Balkan breeds. Nearly all the British breeds clustered together, and several were represented by long branches typically seen in isolated populations comprising a small population size. Closely related breeds were represented by a common branch originating from the same basal node and finally diverging into several short branches (2 or 3 branches). Notably, some Belgian breeds (Flemish Red and Blanc Blue Belge) known to have a British ancestral origin in addition to other composite French breeds (Bretonne Black Pied, Pie Rouge des Plaines, and French Red Pied Lowland) were included in the first group. Most Iberian breeds clustered together and had relatively short branches, which may reflect their geographic isolation and low degree of divergence^21^. Of note, the Spanish Pasiega and Terrana breeds grouped with the British and Holstein group, suggesting an exotic admixture. Similarly, and in agreement with previous reports^15,26^, the contribution of foreign breeds from different origins to the development of Russian cattle was reflected in the dispersal of Russian breeds between several of the aforementioned groups (Fig. 8). Balkan populations were represented by short branches, indicative of a common ancestry and a high genetic diversity. Jersey and Guernsey breeds grouped with several Albanian populations, coinciding with a massive gene flow resulting from the known replacement crossing with the Jersey breed in Albania and Bulgaria^7^. The Neighbor-Net with its narrow reticulation and the common branch that originated from the same basal node demonstrated the close relationship and co-evolution of several breeds.

**Figure 9:**
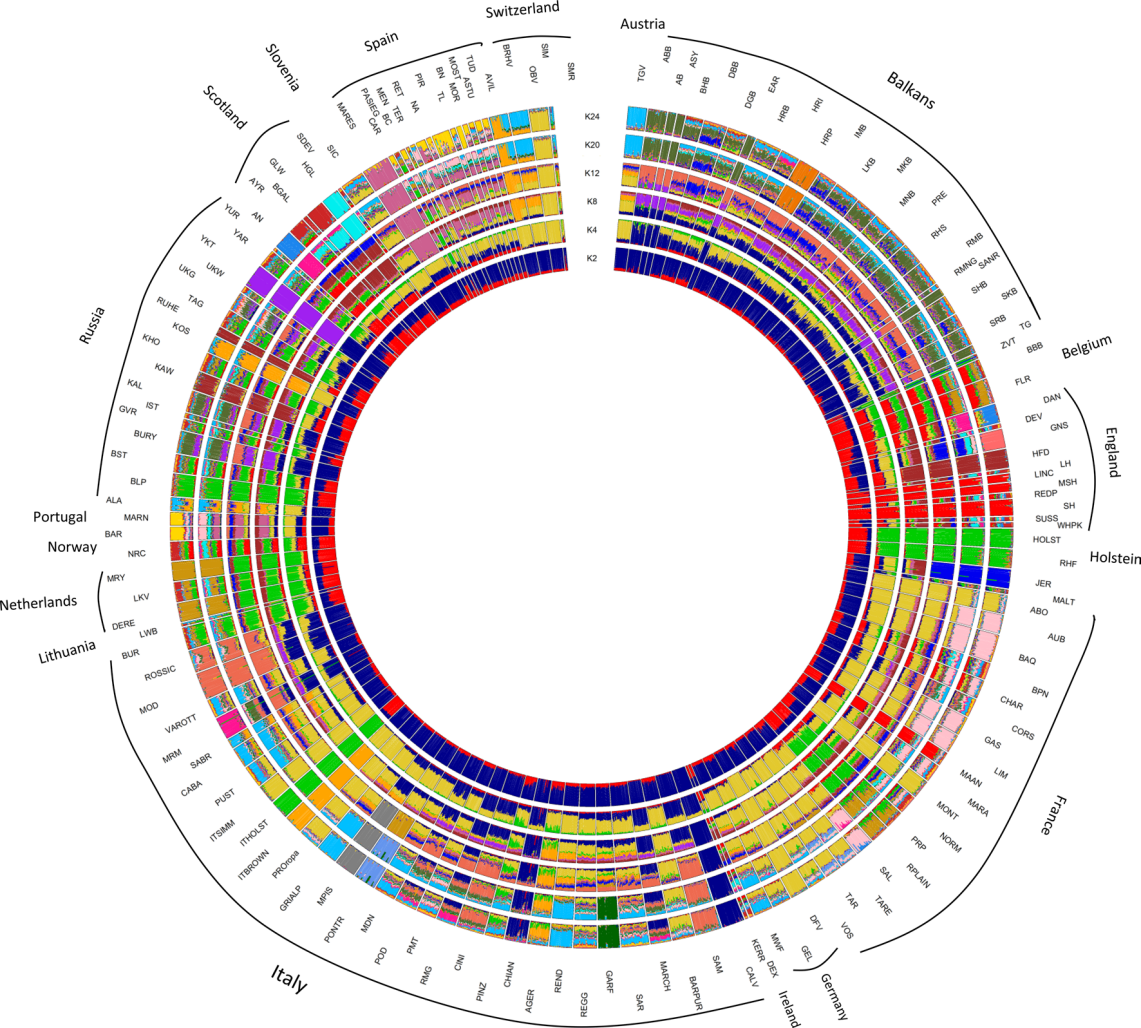
Circular representation of the population structure for the 147 European cattle breeds inferred from the ADMIXTURE analysis. The tested numbers of clusters (K) assumed in the total sample were K = 2, 4, 8, 12, 20 and 24. For full definition of breeds, see Table S1.

In the ADMIXTURE analysis of the European breeds (Fig. 9), at low K values (from 2 to 8), some cosmopolitan breeds under strong artificial selection, such as Holstein, as well as the divergent breeds described above (Yakut, in violet at K = 8), showed a clear separation, consistent with the MDS results. When K increased from 8 to 24, breeds and groups were progressively assigned to separate clusters. The breeds belonging to the Balkan group continued to show high levels of admixture at a high K value (K = 24), with some exceptions. Indeed, at K = 20, a breed-specific cluster was observed for Croatian Podolian (orange in Balkans). At K = 24, several European breeds formed a distinct cluster: Tyrolean Grey (Austria), Hereford (England), Jersey, and some breeds from Tuscany (Pontremolese and Mucca Pisana). The greater differentiation for these breeds could be related to a combination of inbreeding and genetic divergence due to an isolated breeding history.

#### A focus on the Italian breeds in a European context

In a European context (Figs. 7 and 8), we identified two major groups of Italian breeds (Northern-Central and Podolian-derived breeds) based on their historical origin and degree of sharing of genomic components. This result was consistent with that of a recent study by our group^17^, as well as with the mentioned above results reported on a global level. The Northern Italian breeds (e.g., Rendena, Pezzata Rossa d’Oropa, Pustertaler, and Cabannina) were genetically close to several European breeds of the Alpine area (Red Pied breeds and Brown Cattle), suggesting a contribution of the central European gene pool to the composition of these breeds. Indeed, spotted, brown, and grey Alpine cattle have influenced several Northern-Italian breeds^4^. The oldest known herdbook for cattle dates from between 1775 and 1782, and originated from the Swiss canton of Schwyz where the gray-brown mountain cattle (Braunvieh) evolved. In 1875, Schwyz cattle and two Braunvieh populations were recognized and combined as the Swiss Brown, comprising a common herdbook; these cattle were the ancestors of several Alpine, Italian, and Spanish brown cattle^4^.

Northern-Central Italian populations (Piedmontese, Garfagnina, Mucca Pisana, Reggiana, Modenese, and Pontremolese) tended to cluster with Iberian and some French breeds (Maraichine and Charolaise) (Fig. 7). It has been posited that Iberian breeds are the result of introgression of Africa cattle into the local European cattle and do not have indicine ancestry, similar to that seen for some French (Maraichine, Gascon, Limousin) and Piedmontese breeds^10^. Our results supported this hypothesis, with the absence of indicine introgression in Iberian breeds (Fig. 6). Our findings (Figs. 7 and 8) also revealed genetic proximity between the Italian cattle belonging to the Podolian group (Italian beef cattle and the Sicilian breeds) and several Balkan populations, such as the Croatian Podolian and Buša strains. Mitochondrial DNA analysis has shown that Italian and Balkan cattle differ in haplogroup distribution^33^, indicating that the maternal lineages are of local descent and that the Podolian gene flow into Italy was male-mediated. In our study, with the inclusion of additional local breeds, we found that a close relationship exists between Buša populations and several Italian breeds. A recent study, although on a smaller number of breeds, noted that these breeds received a similar contribution from African taurine and indicine cattle and subsequently evolved independently^14^. Analysis of the European dataset also confirmed the Podolian origins of Sicilian cattle, which had recently been classified as non-Podolian breeds^29^. We also noted the outlier behavior of some Italian breeds, such as that of Burlina cattle. Neighbor-Net and MDS analysis separated this breed from the Northern Italian cluster owing to admixture events with Italian Holstein cattle^17,34^. Finnish Ayrshire, Norwegian Red, and Kholmogory were the European breeds closest to Burlina cattle and the breeds that were likely to have been influenced by the Holstein genetic component.

## Conclusions

In this study, we have assembled a large cattle dataset to refine and extend previous studies and to understand better the genetic origins of European cattle, with a particular focus on Italian breeds. The genome ROH coverage differs within and among breeds and subspecies, and reflects the complex breeding history of cattle. Our findings indicated that, when compared with cattle worldwide, some breeds from Tuscany show high levels of inbreeding. In addition to confirming previous reports, our results also showed new insights into the complex origin of some Italian breeds. In a worldwide context with other breeds, the Italian cattle clustered with other European and Asian taurine breeds. Overall, the grouping of the breeds on the Neighbor-Net network was consistent with their geographic origins. The genetic diversity of European cattle is described by a gradient of distribution along three main directions. Within the European context, we identified two main groups for the Italian breeds based on their historical origin and degree of conservation of ancestral genomic components. In this study, we also observed that some Italian breeds not included in previous studies, such as the Sicilian breeds, experienced a non-European influence in the past, similar to that observed for Podolian-derived breeds. A common genomic component between Balkan and several Italian cattle breeds (beef breeds and Sicilian cattle) was also revealed. However, further study will be necessary to test and validate our results.

## Methods

### Data merging and filtering

For analyses of comparative population genomics, SNP genotyping data from previously published^7,10,12,15–17,21,24^ work were merged with PLINK^34^. Spanish breeds (Terrana, Asturiana, Pasiega, and Tudanca) were genotyped for this study. Detailed information about all the breeds and samples is shown in Table S1. All individuals were genotyped using the Bovine SNP50K BeadChip. A series of quality control procedures were performed. Breeds with fewer than three samples were removed. To reduce the bias from over-represented breeds, data were restricted to a maximum of 30 animals per breed, selected at random. Only markers located on autosomes were considered. First, SNPs with a minor allele frequency (MAF) lower than 0.05 and call rate lower than 95%, as well as poorly genotyped individuals (call rate < 90%), were removed, resulting in a dataset comprising a total of 3,283 individuals and 205 populations. For easier comparison and to simplify the graphic representation, the individuals were first labeled as belonging to different groups according to subspecies and geographic origin (Table 1 and Fig. 1). Moreover, to better explore the genetic relationships among European cattle, with a particular focus on Italian breeds, a reduced dataset containing 147 breeds and 2,498 individuals was also created.

### Runs of homozygosity

For this analysis, breeds with less than 10 animals (Supplementary Table 1) were excluded to avoid unbiased estimates resulting from a low number of individuals per breed. Runs of homozygosity were detected as described by Mastrangelo et al.^17^, using a sliding window approach of 50 SNPs in PLINK v.1.07^35^. The minimum length that constituted a ROH was set to 4 Mb. Moreover, (i) one missing SNP was allowed in the ROH and up to one possible heterozygous genotype; (ii) the minimum number of consecutive SNPs that constituted a ROH was set to 50; (iii) the minimum density was set at 1 SNP every 100 kb; and (iv) 1 Mb was set as the maximum gap between consecutive SNPs. The genomic inbreeding coefficient (*F*_ROH_) was calculated for each breed.

### Genetic relationships and admixture

Pairwise genetic relationships were estimated using a matrix of genome-wide identity-by-state (IBS) genetic distances calculated by PLINK^35^ and plotted using a MDS plot in the R environment.

To assess reticulated relationships between populations, the ARLEQUIN software^36^ was used to estimate Reynolds genetic distances and neighbor networks were constructed from the estimated genetic distances using SPLITSTREE^37^.

Population structure was assessed by the maximum likelihood-based approach implemented in the ADMIXTURE software v1.3.0^38^ by applying the default settings. Different K values with the mixed ancestry model (K = 2 to 50) were estimated to examine patterns of ancestry and admixture in the dataset. The BITE R package^39^ was used to graphically represent the results. All these analyses were conducted on both datasets, i.e., for both the global and European breeds.

Finally, the THREEPOP program implemented in Treemix^40^ was run to verify whether populations suspected of being admixed indeed showed significant signatures of admixture. This program calculated *f*3 statistics for all possible triplets from the selected populations. If population A was a mixture of two other populations, B and C, the *Z*-score computed for each tested triplet would have a significant negative value.

## Supplementary information

Supplementary Table S1.

Supplementary Table S2.

Supplementary Figure S1.

Supplementary Figure S2.

Supplementary information.

## Data Availability

The authors confirm that the data supporting the findings of this study are available within the article and its supplementary materials. The raw genetic datasets generated during the current study are available from the corresponding author on reasonable request.
