# Supplementary material for: Refining the genetic structure and relationships of European cattle breeds through meta-analysis of worldwide genomic SNP data, focusing on Italian cattle
Source: Sci Rep. 2020 Sep 3;10:14522. doi: 10.1038/s41598-020-71375-2 (PMC7471305; doi:10.1038/s41598-020-71375-2)
Supplement: Supplementary file 3 — Supplementary Figure S1. [file 41598_2020_71375_MOESM3_ESM.pdf]

Refining the genetic structure and relationships of European cattle breeds through meta-analysis of worldwide genomic SNP data, focusing on Italian cattle

Salvatore Mastrangelo[1], Marco Tolone[1], Slim Ben Jemaa[2], Gianluca Sottile[3], Rosalia Di Gerlando[1], Oscar Cortés[4], Gabriele Senczuk[5], Baldassare Portolano[1], Fabio Pilla[5] and Elena Ciani[6]

[1]Dipartimento Scienze Agrarie, Alimentari e Forestali, University of Palermo, 90128 Palermo, Italy.
[2]Laboratoire des Productions Animales et Fourragères, Institut National de la Recherche Agronomique de Tunisie, Université de Carthage, 2049 Ariana, Tunisia.
[3]Dipartimento Scienze Economiche, Aziendali e Statistiche, University of Palermo, 90128 Palermo, Italy.
[4]Departamento de Produccion Animal, Universidad Complutense de Madrid, 28040 Madrid, Spain
[5]Dipartimento di Agricoltura, Ambiente e Alimenti, University of Molise, 86100 Campobasso, Italy
[6]Dipartimento di Bioscienze Biotecnologie e Biofarmaceutica, University of Bari, 70124 Bari, Italy.

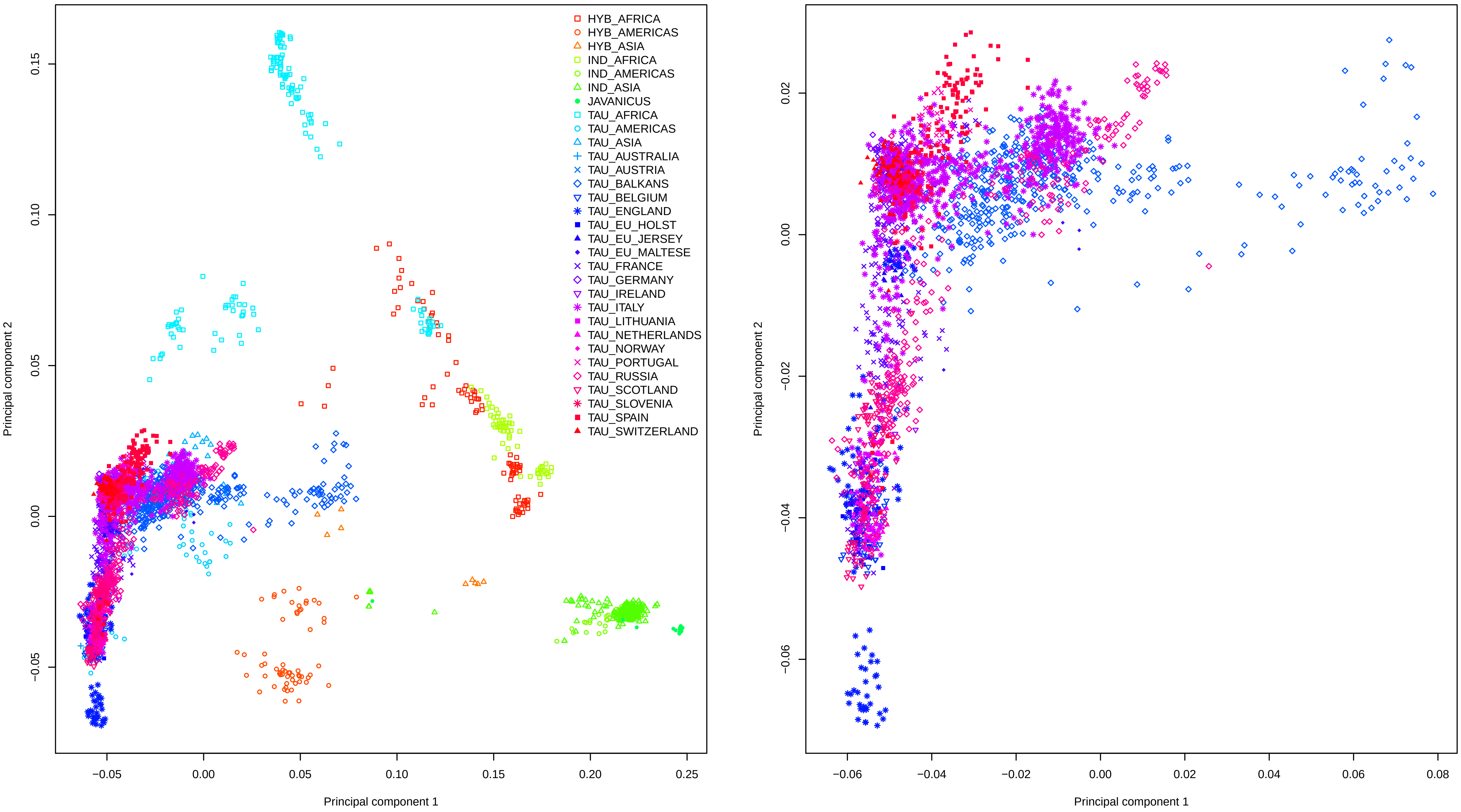

HYB_AFRICA
HYB_AMERICAS
HYB_ASIA
IND_AFRICA
IND_AMERICAS
IND_ASIA
JAVANICUS
TAU_AFRICA
TAU_AMERICAS
TAU_ASIA
TAU_AUSTRALIA
TAU_AUSTRIA
TAU_BALKANS
TAU_BELGIUM
TAU_ENGLAND
TAU_EU_HOLST
TAU_EU_JERSEY
TAU_EU_MALTESE
TAU_FRANCE
TAU_GERMANY
TAU_IRELAND
TAU_ITALY
TAU_LITHUANIA
TAU_NETHERLANDS
TAU_NORWAY
TAU_PORTUGAL
TAU_RUSSIA
TAU_SCOTLAND
TAU_SLOVENIA
TAU_SPAIN
TAU_SWITZERLAND
Principal component 1
Principal component 2
