# Supplementary material for: Refining the genetic structure and relationships of European cattle breeds through meta-analysis of worldwide genomic SNP data, focusing on Italian cattle
Source: Sci Rep. 2020 Sep 3;10:14522. doi: 10.1038/s41598-020-71375-2 (PMC7471305; doi:10.1038/s41598-020-71375-2)
Supplement: Supplementary file 4 — Supplementary Figure S2. [file 41598_2020_71375_MOESM4_ESM.pdf]

Refining the genetic structure and relationships of European cattle breeds through meta-analysis of worldwide genomic SNP data, focusing on Italian cattle

Salvatore Mastrangelo[1], Marco Tolone[1], Slim Ben Jemaa[2], Gianluca Sottile[3], Rosalia Di Gerlando[1], Oscar Cortés[4], Gabriele Senczuk[5], Baldassare Portolano[1], Fabio Pilla[5] and Elena Ciani[6]

[1]Dipartimento Scienze Agrarie, Alimentari e Forestali, University of Palermo, 90128 Palermo, Italy.
[2]Laboratoire des Productions Animales et Fourragères, Institut National de la Recherche Agronomique de Tunisie, Université de Carthage, 2049 Ariana, Tunisia.
[3]Dipartimento Scienze Economiche, Aziendali e Statistiche, University of Palermo, 90128 Palermo, Italy.
[4]Departamento de Produccion Animal, Universidad Complutense de Madrid, 28040 Madrid, Spain
[5]Dipartimento di Agricoltura, Ambiente e Alimenti, University of Molise, 86100 Campobasso, Italy
[6]Dipartimento di Bioscienze Biotecnologie e Biofarmaceutica, University of Bari, 70124 Bari, Italy.

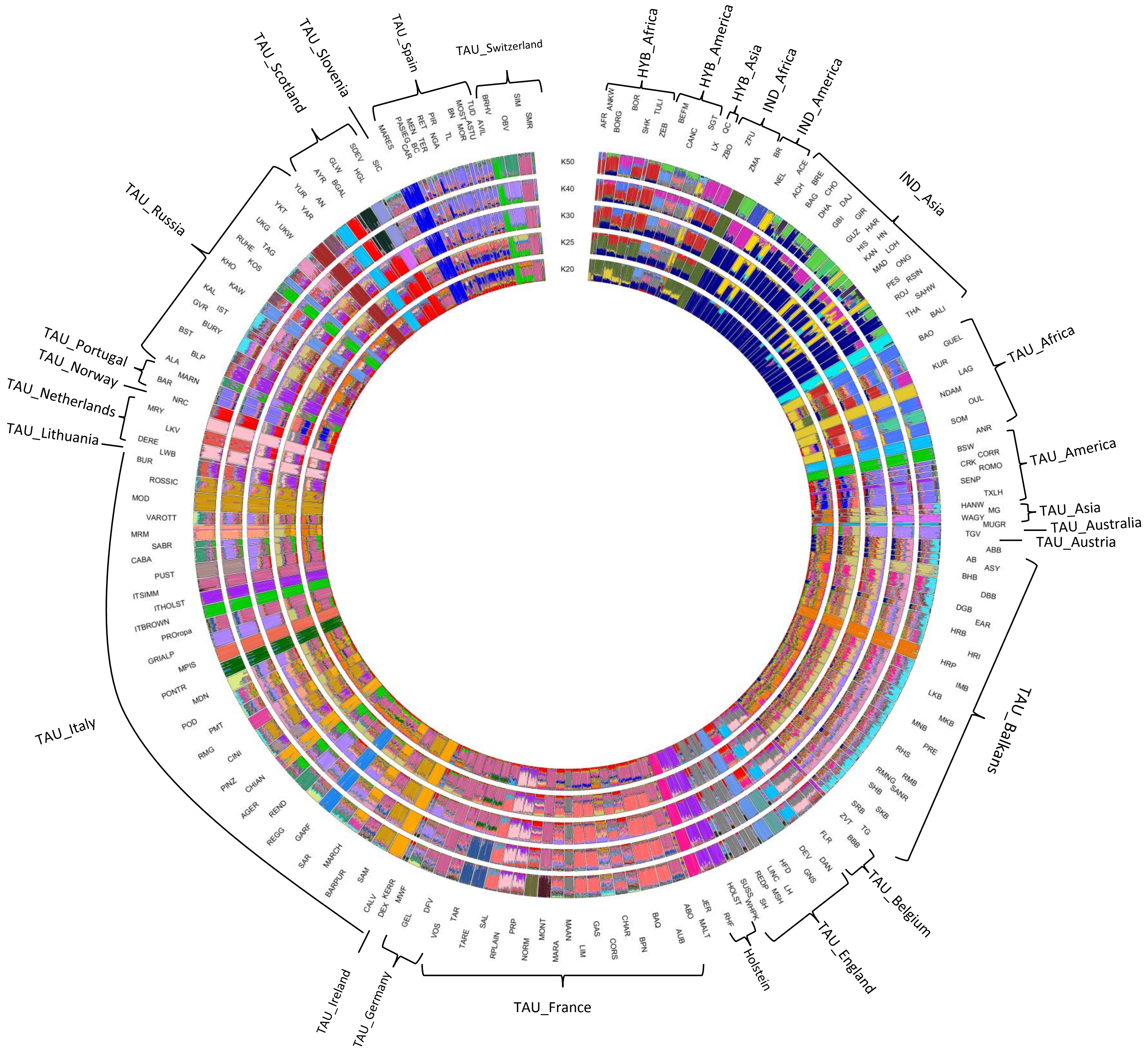

TAU_Switzerland
TAU_Spain
TAU_Slovenia
TAU_Scotland
TAU_Russia
TAU_Portugal
TAU_Norway
TAU_Netherlands
TAU_Lithuania
TAU_Italy
TAU_Ireland
TAU_Germany
TAU_France
Holstein
TAU_England
TAU_Belgium
TAU_Balkans
TAU_Austria
TAU_Australia
TAU_Asia
TAU_America
TAU_Africa
IND_Asia
IND_America
IND_Africa
HYB_Asia
HYB_America
HYB_Africa
K50
K40
K30
K25
K20
AFR ANKW BORG BOR SHK TULI ZEB
BEFM CANC SGT LX QC ZBO
ZFU ZMA BR NEL
ACE ACH BRE BAG CHO DHA DAJ GBI GIR GUZ HAR HIS HN KAN LOH MAD ONG PES RSIN ROJ SAHW THA BALI
BAO GUEL KUR LAG NDAM OUL SOM
ANR BSW CORR CRK ROMO SENP TXLH
HANW MG WAGY MUGR
TGV
ABB AB ASY BHB DBB DGB EAR HRB HRI HRP IMB LKB MKB MNB PRE RHS RMB RMNG SANR SHB SKB SRB TG ZVT BBB
FLR DEV DAN GNS HFD LINC LH REDP MSH SH SUSS WHPK HOLST RHF
JER ABO MALT AUB BAQ BPN CHAR CORS GAS LIM MAAN MARA MONT NORM PRP RPLAIN SAL TARE TAR VOS DFV
GEL MWF KERR DEX CALV
SAM BARPUR MARCH SAR GARF REGG REND AGER CHIAN PINZ CINI RMG PMT POD MDN PONTR MPIS GRIALP PROropa ITBROWN ITHOLST ITSIMM PUST CABA SABR MRM VAROTT MOD ROSSIC BUR LWB DERE
LKV MRY
NRC
MARN BAR ALA
BLP BST BURY GVR IST KAL KAW KHO KOS RUHE TAG UKG UKW YKT YAR YUR AN AYR
BGAL GLW HGL SDEV
SIC
MARES PASIEGCAR MEN BC RET TER PIR NGA BN TL MOST MOR TUD ASTU
AVIL BRHV OBV SIM SMR
