# Supplementary material for: Refining the genetic structure and relationships of European cattle breeds through meta-analysis of worldwide genomic SNP data, focusing on Italian cattle
Source: Sci Rep. 2020 Sep 3;10:14522. doi: 10.1038/s41598-020-71375-2 (PMC7471305; doi:10.1038/s41598-020-71375-2)
Supplement: Supplementary file 5 — Supplementary information. [file 41598_2020_71375_MOESM5_ESM.pdf]

Refining the genetic structure and relationships of European cattle breeds through meta-analysis of worldwide genomic SNP data, focusing on Italian cattle

Salvatore Mastrangelo[1], Marco Tolone[1], Slim Ben Jemaa[2], Gianluca Sottile[3], Rosalia Di Gerlando[1], Oscar Cortés[4], Gabriele Senczuk[5], Baldassare Portolano[1], Fabio Pilla[5] and Elena Ciani[6]

[1]Dipartimento Scienze Agrarie, Alimentari e Forestali, University of Palermo, 90128 Palermo, Italy.

[2]Laboratoire des Productions Animales et Fourragères, Institut National de la Recherche Agronomique de Tunisie, Université de Carthage, 2049 Ariana, Tunisia.

[3]Dipartimento Scienze Economiche, Aziendali e Statistiche, University of Palermo, 90128 Palermo, Italy.

[4]Departamento de Produccion Animal, Universidad Complutense de Madrid, 28040 Madrid, Spain

[5]Dipartimento di Agricoltura, Ambiente e Alimenti, University of Molise, 86100 Campobasso, Italy

[6]Dipartimento di Bioscienze Biotecnologie e Biofarmaceutica, University of Bari, 70124 Bari, Italy.

Supplementary information

Table S1 Name of the breeds, breed codes, sample size, sub-species, continent and geographic origin, and source of genotyping data.

Table S2 Results of the significant *f3* test among the worldwide cattle breeds in this study. For full definition of breeds see Table S1.

Figure S1 Genetic relationships among the a) worldwide cattle breeds and b) European breeds as inferred by MDS analysis. The breeds were grouped according to their geographical origins and sub-species.

Figure S2 Circular representation of the worldwide population structure for the 205 different domesticated bovid breeds inferred from the ADMIXTURE analysis. The tested numbers of clusters (K) assumed in the total sample were K = 20, 25, 30, 40 and 50.
